# Instrumental variable estimation of truncated local average treatment effects

**DOI:** 10.1371/journal.pone.0249642

**Published:** 2021-04-05

**Authors:** Byeong Yeob Choi

**Affiliations:** Department of Population Health Sciences, UT Health San Antonio, San Antonio, TX, United States of America; University of South Florida, UNITED STATES

## Abstract

Instrumental variable (IV) analysis is used to address unmeasured confounding when comparing two nonrandomized treatment groups. The local average treatment effect (LATE) is a causal estimand that can be identified by an IV. The LATE approach is appealing because its identification relies on weaker assumptions than those in other IV approaches requiring a homogeneous treatment effect assumption. If the instrument is confounded by some covariates, then one can use a weighting estimator, for which the outcome and treatment are weighted by instrumental propensity scores. The weighting estimator for the LATE has a large variance when the IV is weak and the target population, i.e., the compliers, is relatively small. We propose a truncated LATE that can be estimated more reliably than the regular LATE in the presence of a weak IV. In our approach, subjects who contribute substantially to the weak IV are identified by their probabilities of being compliers, and they are removed based on a pre-specified threshold. We discuss interpretation of the proposed estimand and related inference method. Simulation and real data experiments demonstrate that the proposed truncated LATE can be estimated more precisely than the standard LATE.

## Introduction

Instrumental variable (IV) analysis can be used to address bias from unobserved confounding in non-randomized studies when estimating a treatment effect on an outcome of interest. Many IV methods have been developed based on linear and survival regression models [1–5], however, they require a homogeneous treatment effect assumption to find meaningful causal effects in the framework of the Rubin causal model [6, 7]. This homogeneity assumption may be too strong for application to real observational studies. Imbens and Angrist [8] introduced a causal estimand that can be identified by an IV called the local average treatment effect (LATE). This estimand does not require treatment effects to be homogeneous for all study subjects. The LATE represents the average treatment effect for a group of compliers, who choose their treatment according to the random variation in an IV.

A key assumption for a valid IV is that it is independent of the potential outcomes and treatments. Angrist et al. [9] showed that if this assumption holds without any covariates, then the Wald estimator, the ratio of sample covariances, is consistent for the LATE. Sometimes, IVs are considered to be random only if some covariates are controlled. For example, Brooks et al. [10] controlled for patient’s socioeconomic and clinical characteristics, and nearest hospital distance in their IV analysis, where breast conserving surgery plus irradiation (BCSI) rate was used as an IV to compare BCSI and mastectomy in stage II breast cancer patients. Adjusting for covariates to generate valid IVs is straightforward in IV linear regression models, such as two-stage least squares. However, covariate adjustment in estimation of the LATE requires different approaches. Abadie [11] developed a method to estimate the LATE conditional on observed covariates. This approach requires the estimation of instrumental propensity scores (IPSs), which are analogous to propensity scores (PSs) for the probability of treatment, and the construction of an outcome model for compliers as a function of the treatment and covariates, which is called the local average response function. Weighting and regression methods have been developed to estimate the conditional and marginal LATEs, which address confounded instruments [12, 13]. In this study, we particularly focused on the weighting approach, which only requires the IPSs to be estimated.

In general, PS weighting requires a positivity assumption that the PSs are between 0 and 1. Extreme PSs close to 0 or 1 are problematic because the average treatment effect (ATE) is estimated from a pseudo-population, where each subject has been treated and untreated. Subjects with extreme PSs receive extremely large PS weights to realize the pseudo-population. Extreme PSs result in limited overlap of covariate distributions between comparison groups and make the inverse probability weighting (IPW) estimator unstable. Methods such as truncation and overlap weights have been proposed to deal with this problem. Truncation [14] removes subjects whose PSs are extreme, say below 0.05 or above 0.95. Overlap weights [15] continuously down-weight the subjects with extreme PSs rather than discarding them. Various simulation studies have showed that these methods perform much better than the IPW estimator [16, 17].

Weak IVs are known to amplify the bias of IV estimators and complicate the related asymptotic distributions [18–22]. To deal with weak instruments, we propose a truncation method for the weighting estimator of the LATE. For this, we use the lemma of Abadie [11] that the probabilities of being a complier are greater than 0 for all subjects when the monotonicity assumption of Angrist et al. [9] holds. Therefore, we seek more reliable IV estimation of the weighting estimator for the LATE by truncating the subjects whose probabilities of being a complier are very close to or below zero. The proposed method follows the framework of the truncation method in PS analysis, but it tackles the weak IV problem and related positivity issue that occur when weighting study subjects to identify the population of compliers.

The remainder of this article is organized as follows. We begin with a discussion of the notation and assumptions used for the LATE with a confounded IV. Then, we present the main results for the proposed truncated local average treatment effect (TLATE), which can be estimated more reliably than the LATE when the IV is weak. Based on simulations, we compare the proposed TLATE estimator with the LATE estimator in terms of variance and IV strength. For real-world application, we used the data from a population-based prospecitive cohort study to estimate the effect of a respiratory syncytial virus on lower respiratory tract infections. We end the article with concluding remarks.

## Notation and assumptions

To define the LATE, we introduce some notation and assumptions. Let *Z* ∈ {0, 1} be the binary IV and *D*(*z*) ∈ {0, 1} be the binary potential treatment value that would be seen if *Z* = *z*. The actual treatment received is defined as *D* = (1 − *Z*)*D*(0) + *ZD*(1). Let *Y*(*z*, *d*) be the potential outcome that would be seen if *Z* = *z* and *D* = *d*. Under the assumption of exclusion restriction (Assumption 3 below), *Y*(*z*, *d*) can be written as *Y*(*d*). Then, the observed outcome can be expressed as *Y* = *DY*(1) + (1 − *D*)*Y*(0). Let *X* be a vector of observed covariates.

Angrist et al. [9] divided the population into four groups in terms of *D*(0) and *D*(1): compliers if *D*(1) > *D*(0), always-takers if *D*(1) = *D*(0) = 1, never-takers if *D*(1) = *D*(0) = 0 and defiers if *D*(1) < *D*(0). These groups can only be partially identified because only *D*(0) or *D*(1) is observed for each subject. Among these four groups, under certain assumptions, causal treatment effects are identifiable only for compliers. Let *U* denote the latent compliance class, where *U* = 0 for never-takers, *U* = 1 for always-takers and *U* = 2 for compliers. Defiers are removed under the assumption of monotonicity (Assumption 5 below).

Angrist et al. [9] defined the LATE as

$$\theta^{c}=E\left\{Y\left(1\right)-Y\left(0\right)|U=2\right\}.$$

If the instrument is strongly ignorable without *X*, then *θ*^*c*^ is estimated by the Wald estimator. If strongly ignorable instrument assignment requires conditioning on *X*, then the Wald estimator is not valid for *θ*^*c*^. To get a valid IV estimator for *θ*^*c*^ in such a case, we must employ an IPS, defined as *e*(*X*) = pr(*Z* = 1 ∣ *X*) [11, 12, 23]. We adapt the following assumptions of Abadie [11] and Frölich [13] for use in the IPS.

**Assumption 1** *Independence of the instrument*:

Z⊥{Y(0,0),Y(0,1),Y(1,0),Y(1,1),D(0),D(1)}|X,

where ⊥ denotes statistical independence.

**Assumption 2** *Positivity*: 0 < *e*(*X*) < 1.

**Assumption 3** *Exclusion restriction*: *Y*(0, *d*) = *Y*(1, *d*) *for* *d* = {0, 1}.

**Assumption 4** *Nonzero average causal effect of* *Z* *on* *D*: *pr*{*D*(1) = 1 ∣ *X*} > *pr*{*D*(0) = 1 ∣ *X*}.

**Assumption 5** *Monotonicity*: *pr*{*D*(1) ≥ *D*(0) ∣ *X*} = 1.

Assumption 1 means that *Z* is as-if randomized once we condition on *X*. Assumption 2 means that any study subject has a positive probability of being assigned to both instrument groups. Assumption 3 means that variation in *Z* affects the potential outcomes only through its effect on *D*. Assumption 4 indicates that *Z* is positively correlated with *D* given *X*. Assumption 5 excludes defiers from the study population. Monotonicity trivially holds when only the participants assigned to a treatment arm have the opportunity to receive the active treatment, as in a single consent design [24].

To estimate the LATE with covariates, one can use the weighting IV estimator presented in equation (11) of Frölich [13]:

$$\begin{array}{c} \{\sum_{i=1}^{n}\frac{Z_{i}Y_{i}}{\hat{e}\left(X_{i}\right)}-\sum_{i=1}^{n}\frac{\left(1-Z_{i}\right)Y_{i}}{1-\hat{e}\left(X_{i}\right)}\}/\{\sum_{i=1}^{n}\frac{Z_{i}D_{i}}{\hat{e}\left(X_{i}\right)}-\sum_{i=1}^{n}\frac{\left(1-Z_{i}\right)D_{i}}{1-\hat{e}\left(X_{i}\right)}\}, \end{array}$$ (1)

where $\hat{e}\left(X_{i}\right)$ is the consistently estimated IPS for subject *i*. We can see that the numerator and denominator of estimator (1) are the IPW estimators for *E*{*Y*(*D*(1), 1) − *Y*(*D*(0), 0)} and *E*{*D*(1) − *D*(0)}, which represent the average causal effects of the instrument on the outcome and treatment, respectively. By Proposition 1 in Angrist et al. [9], *θ*^*c*^ is the ratio of these two average treatment effects. Therefore, estimator (1) is a consistent estimator for *θ*^*c*^.

## Truncated local average treatment effects

Instead of estimator (1), one may want to use the following normalized estimator:

$$\begin{array}{c} {\hat{\theta }}^{c}=\frac{{\left(\sum_{i=1}^{n}\frac{Z_{i}}{\hat{e}\left(X_{i}\right)}\right)}^{-1}\sum_{i=1}^{n}\frac{Z_{i}Y_{i}}{\hat{e}\left(X_{i}\right)}-{\left(\sum_{i=1}^{n}\frac{1-Z_{i}}{1-\hat{e}\left(X_{i}\right)}\right)}^{-1}\sum_{i=1}^{n}\frac{\left(1-Z_{i}\right)Y_{i}}{1-\hat{e}\left(X_{i}\right)}}{{\left(\sum_{i=1}^{n}\frac{Z_{i}}{\hat{e}\left(X_{i}\right)}\right)}^{-1}\sum_{i=1}^{n}\frac{Z_{i}D_{i}}{\hat{e}\left(X_{i}\right)}-{\left(\sum_{i=1}^{n}\frac{1-Z_{i}}{1-\hat{e}\left(X_{i}\right)}\right)}^{-1}\sum_{i=1}^{n}\frac{\left(1-Z_{i}\right)D_{i}}{1-\hat{e}\left(X_{i}\right)}}, \end{array}$$ (2)

where the numerator and denominator are the Hajek estimators for the average treatment effects of the instrument on the outcome and treatment, respectively. The weighting estimator for the LATE, ${\hat{\theta }}^{c}$, is obtained from the identification result in Theorem 1 of Frölich [13]:

$$\begin{array}{c} \theta^{c}=\int \theta^{c}\left(x\right)f^{c}\left(x\right)dx=\frac{\int \tau (x)f(x)dx}{\int \delta (x)f(x)dx}, \end{array}$$ (3)

where *f*^*c*^(*x*) = pr(*X* = *x* ∣ *U* = 2) is the density function of *X* for compliers, *f*(*x*) = pr(*X* = *x*) is the marginal density function of *X*, *θ*^*c*^(*x*) = *τ*(*x*)/*δ*(*x*) is the conditional LATE given *x*, and *τ*(*x*) and *δ*(*x*) are the conditional average treatment effects of *Z* on *Y* and *D* given *x*, respectively:

$$\begin{array}{ccc} \theta^{c}\left(x\right) & = & E\{Y(1)-Y(0)|X=x,U=2\}, \end{array}$$

$$\begin{array}{ccc} \tau (x) & = & E\{Y(1,D(1))-Y(0,D(0))|X=x\}, \end{array}$$

$$\begin{array}{ccc} \delta (x) & = & E\{D(1)-D(0)|X=x\}. \end{array}$$

We will call *δ*(*x*) = pr(*U* = 2 ∣ *X* = *x*) the compliance score. The denominator of (3) is *P*(*U* = 2), which is the population size of compliers.

Frölich [13] showed that the semiparametric efficiency bound for *θ*^*c*^ is proportional to 1/*P*(*U* = 2)^2^. Therefore, if the overall proportion of compliers is close to zero, the regression and weighting estimators for the LATE will have very large variances. Abadie [11] showed in Lemma 2.1 of his paper that under monotonicity, *δ*(*x*) is greater than 0, and this positivity assumption implied by monotonicity may be more likely to be violated empirically if *P*(*U* = 2) is very close to zero. That is, subjects whose estimated *δ*(*x*) values are very close to or below zero considerably contribute to a weak instrument. To overcome the problems regarding a weak instrument, we propose the truncated LATE (TLATE), where subjects whose compliance scores are below a pre-specified threshold value are excluded from the analysis.

The proposed estimand is defined as

$$\begin{array}{c} \theta_{T}^{c}=\frac{\int \tau (x)1\{\delta (x)>t\}f(x)dx}{\int \delta (x)1\{\delta (x)>t\}f(x)dx}, \end{array}$$ (4)

where *t* is a pre-specified constant, and 1{⋅} is an indicator function. We consider selecting a value of *t* from the percentiles of the estimated compliance scores, say from the 5th to 50th percentiles, and after truncation with the chosen *t*, the remaining data are used for estimation of the LATE [16]. The TLATE in (4) is different from the LATE in that only the subjects whose probabilities of being a complier are greater than a threshold value *t* contribute to the calculations of the numerator and denominator in (3). By choosing appropriate percentiles, say the 10th or 20th percentile, we can improve the IV strength and variance of the estimate. This is similar to the truncation approach of Crump et al. [14], which excludes subjects whose PSs are close to 0 or 1, improving the variance of the PS weighting estimator. The normalized denominator of (4),

$$\begin{array}{c} \frac{\int \delta (x)1\{\delta (x)>t\}f(x)dx}{\int 1\{\delta (x)>t\}f(x)dx}, \end{array}$$ (5)

is interpreted as the IV strength for the TLATE in the sense that Eq (5) equals the IV strength of the LATE for any *t* smaller than the minimum value of the compliance score.

The following theorem addresses the local causal estimand identified by Eq (4).

**Theorem 1** *Under Assumptions 1-5*, $\theta_{T}^{c}$ *can be expressed as*

$$\begin{array}{c} \theta_{T}^{c}=\frac{\int \theta^{c}\left(x\right)1\left\{\delta \left(x\right)>t\right\}f^{c}\left(x\right)dx}{\int 1\left\{\delta \left(x\right)>t\right\}f^{c}\left(x\right)dx}. \end{array}$$ (6)

Theorem 1 shows that the proposed estimand is obtained as the average of the local average treatment effects of the compliers whose compliance scores are greater than *t*. If *θ*^*c*^(*x*) does not vary by different characteristics *x*, then the TLATE is equivalent to *θ*^*c*^, which is the LATE.

For estimation of the TLATE, following Crump et al. [14], we re-estimate the IPSs for the truncated sample. That is, we select a sample of subjects whose *δ*(*x*) values are greater than a pre-specified *t*. Then, we estimate the IPSs using only this sample and plug the re-estimated IPSs into Eq (2).

To implement our approach, we must estimate *δ*(*x*). In a single consent design, *δ*(*X*_*i*_) = *E*(*D*_*i*_ ∣ *X*_*i*_, *Z*_*i*_ = 1) = pr(*D*_*i*_ = 1 ∣ *X*_*i*_, *Z*_*i*_ = 1). Therefore, we can fit a logistic regression model of *D* on *X* to the group *Z* = 1 and then predict *δ*(*X*_*i*_) for all subjects. This procedure makes all estimated *δ*(*X*_*i*_) values positive. For a general case, we fit both pr(*D*_*i*_ = 1 ∣ *X*_*i*_, *Z*_*i*_ = 1) and pr(*D*_*i*_ = 1 ∣ *X*_*i*_, *Z*_*i*_ = 0) using only the *Z* = 1 and *Z* = 0 groups with logistic regression models. Then, we predict the values of pr(*D*_*i*_ = 1 ∣ *X*_*i*_, *Z*_*i*_ = 1) and pr(*D*_*i*_ = 1 ∣ *X*_*i*_, *Z*_*i*_ = 0) for all subjects and take the differences. This does not guarantee that all estimated *δ*(*X*_*i*_) values are positive. The proposed method can exclude the subjects with negative compliance scores by choosing *t* appropriately.

## Simulation study

We compared the proposed truncation method with the LATE method by evaluating the absolute percentage bias, standard error, and IV strength of the TLATE and LATE estimates using simulated data sets. We considered one covariate *X*, which was a standard normal random variate. Using this *X*, we generated the IV (*Z*) from the following logistic regression model:

$$e\left(x\right)=\text{pr}\left(Z=1|X=x\right)=\frac{\exp(\beta_{0}+\beta_{1}x)}{1+\exp(\beta_{0}+\beta_{1}x)},$$

where *β*_1_ = 1, and *β*_0_ was set such that pr(*Z* = 1) = 0.4. A compliance class *U* took values 0 for never-takers, 1 for always-takers, and 2 for compliers. The compliance score was generated based on the following logistic regression function:

$$\delta \left(x\right)=\text{pr}\left(U=2|X=x\right)=\frac{\exp(\gamma_{0}+\gamma_{1}x)}{1+\exp(\gamma_{0}+\gamma_{1}x)}.$$

The probability of being a never-taker or always-taker was (1 − *δ*(*x*))/2. The value of *γ*_1_ was 1, 2, or, 3, and the value of *γ*_0_ was chosen to implement three scenarios: pr(*U* = 2) = 0.1, 0.3, and 0.5. The treatment variable *D* was then a deterministic function of *Z* and *U*: *D* = *Z*1(*U* = 2)+ *U*1(*U* ≠ 2). We generated the outcome variable *Y* from the following linear model:

Y=D+X+ϵ,

where *ϵ* was a standard normal random variate independent of the other variables. In this setup, the conditional average treatment effects were equal to 1 for all subjects. Thus, the LATE and TLATE were equal to 1. We generated 1000 data sets with a sample size of 1000.

From each simulated data set, we fitted a logistic regression model to estimate the IPSs and calculated the LATE estimate using Eq (2). For the TLATE, we used the 5th to 51st percentiles of the estimated compliance scores for the value of *t*. While increasing the size of the percentile cutpoint, we used a smaller data set. We fitted logistic regression models of *D* on *X* to the *Z* = 1 and *Z* = 0 groups separately. Then, we predicted pr(*D*_*i*_ = 1 ∣ *X*_*i*_, *Z*_*i*_ = 1) and pr(*D*_*i*_ = 1 ∣ *X*_*i*_, *Z*_*i*_ = 0) for all subjects and took the differences as the estimates of the compliance scores.

**Fig 1:**
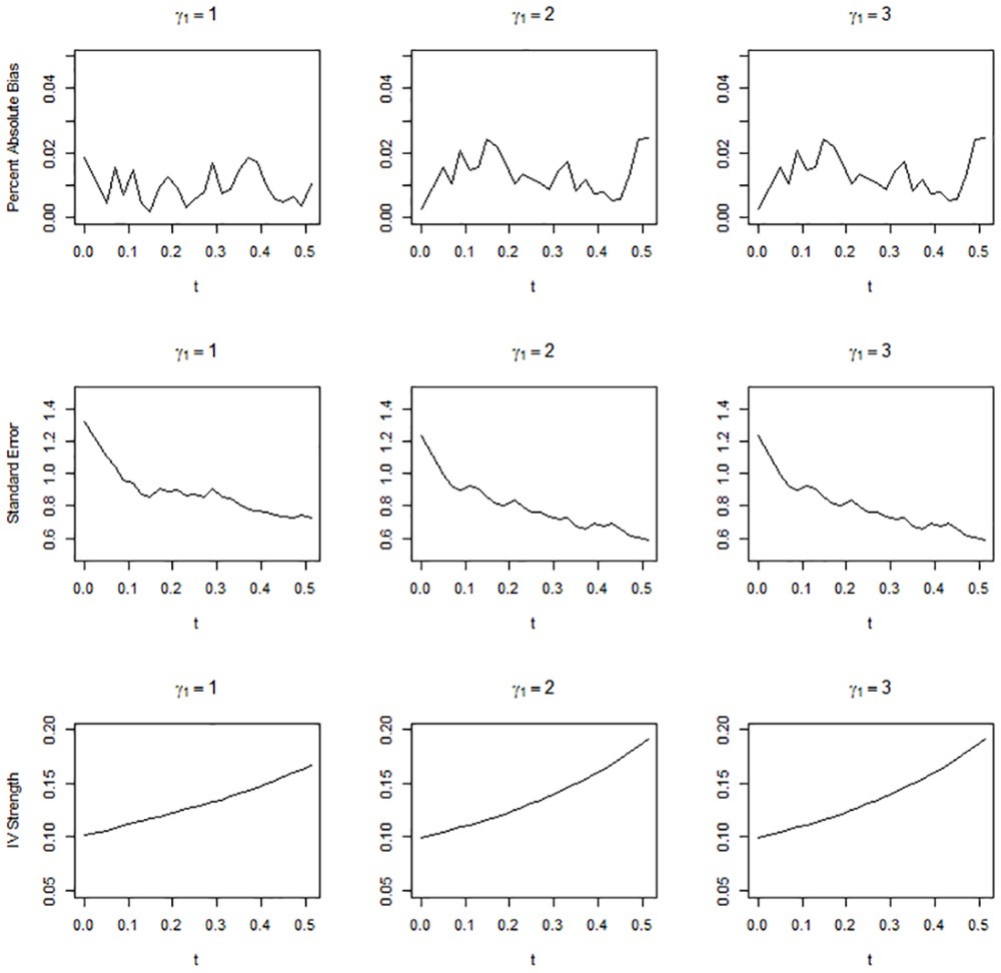
Absolute percentage bias, standard error, and IV strength for the local average treatment effect with a complier population size of 0.1. The X-axis indicates the percentile of the estimated compliance scores for the cutpoint *t*.

**Fig 2:**
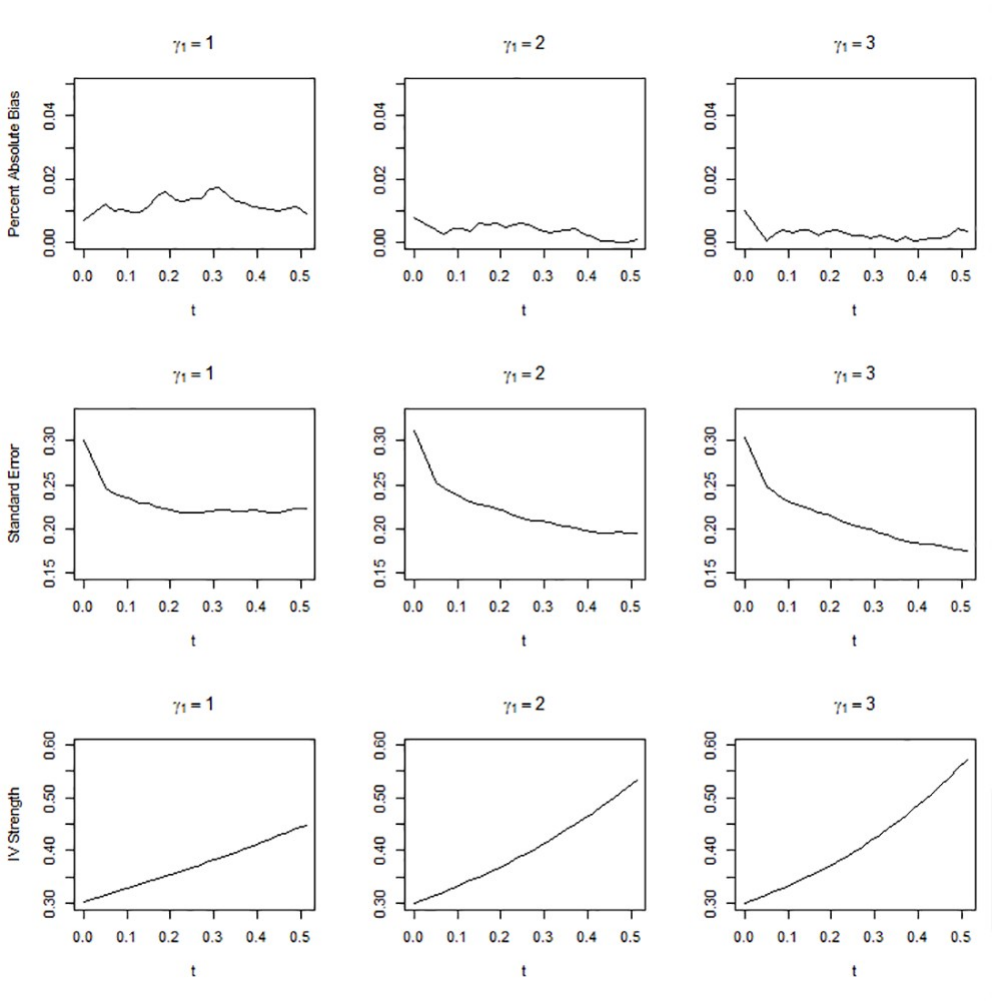
Absolute percentage bias, standard error, and IV strength for the local average treatment effect with a complier population size of 0.3. The X-axis indicates the percentile of the estimated compliance scores for the cutpoint *t*.

**Fig 3:**
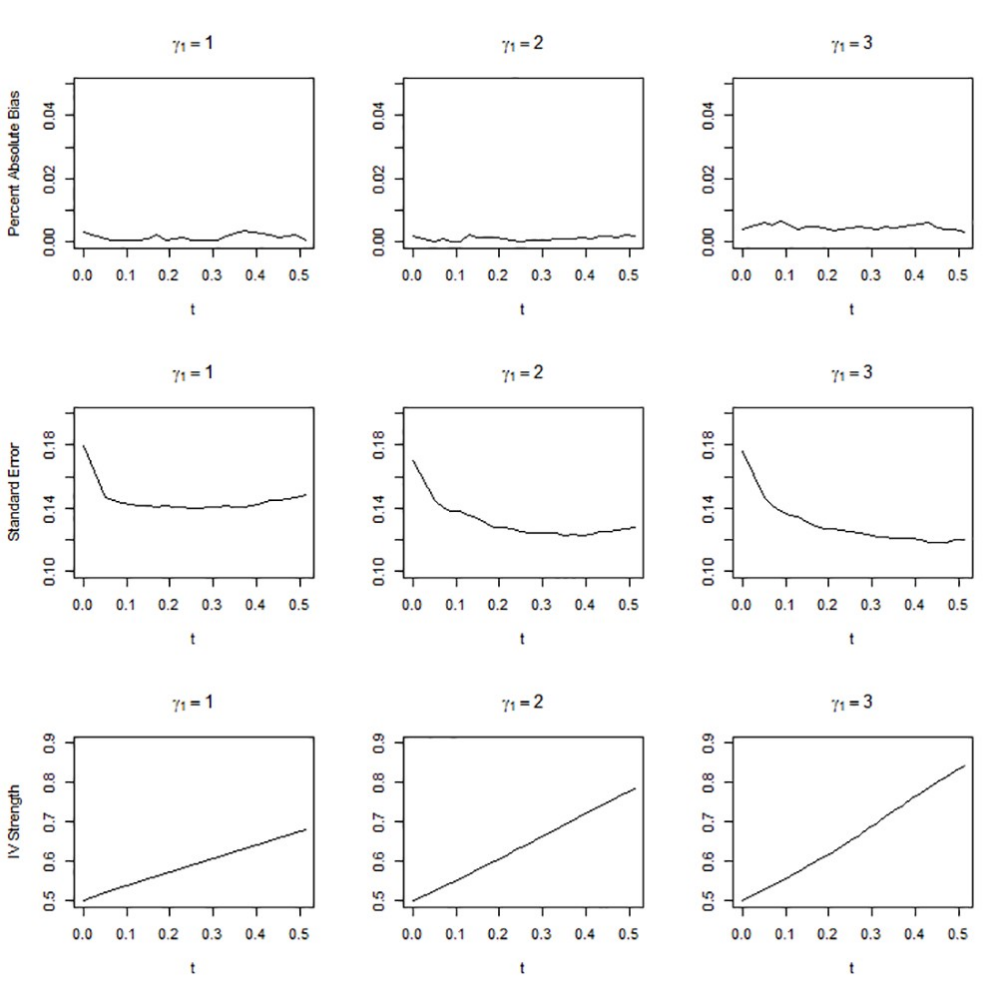
Absolute percentage bias, standard error, and IV strength for the local average treatment effect with a complier population size of 0.5. The X-axis indicates the percentile of the estimated compliance scores for the cutpoint *t*.

Figs 1–3 show the simulation results when the overall IV strength was weak, mild, and moderate, i.e., when pr(*U* = 2) = 0.1, 0.3, and 0.5, respectively. The absolute bias, standard error, and IV strength of the LATE and TLATE estimates are shown as functions of the percentile of the estimated compliance scores for the cutpoint *t*. The first value of each performance measure was obtained without truncation, i.e., when *t* was slightly smaller than the 0th percentile. All estimates had less than 5% bias. The bias was reduced as pr(*U* = 2) increased. In general, as *t* increased, the standard error decreased and the IV strength increased. Truncation became more beneficial as the association between the covariate and compliance class grew: in general, the slopes for the reduction in the standard error and increment in the IV strength became greater as *γ*_1_ increased. The standard error reduced most significantly up to approximately the 10th percentile across the different scenarios of pr(*U* = 2). When *γ*_1_ was 2 or 3, the standard error reduced monotonically up to the 51st percentile. However, when *γ*_1_ = 1, the standard error reached a plateau at approximately the 20th percentile in the scenario of pr(*U* = 2) = 0.3 and at approximately the 10th percentile in the scenario of pr(*U* = 2) = 0.5.

## Application

The PRI.DE study was a population-based prospective cohort study of lower respiratory tract infections (LRTI) in the German population of infants and children aged less than 3 years [25]. Among the 5310 eligible cases of LRTI, Stampf et al. [26] used 3078 complete cases to estimate the marginal odds ratio effect of a respiratory syncytial virus (RSV) on the severity of LRTI. In our study, we used the same data set but estimated the risk difference of severe LRTI.

The outcome was the severity of LRTI, with *Y* = 1 indicating severe LRTI resulting in hospitalization and *Y* = 0 indicating non-severe LRTI requiring only a pediatric practitioner. The treatment variable was current RSV infection, with *D* = 1 indicating the presence of RSV and *D* = 0 otherwise. Among the 3078 patients, 1803 were hospitalized and 1031 were infected with RSV. The covariates included gender, age, external care, siblings, current breast feeding, parental atopy, preterm delivery, tobacco, congenital heart defect, ethnic group, and region of country.

We used former RSV infection as an IV. We assumed former RSV infection did not directly affect the probability of developing severe LRTI during the current RSV infection, and the covariates were given. Our IV was equal to *Z* = 0 if the infant had been infected with RSV formerly and *Z* = 1 if the infant had not. Thus, *D*(0) was the potential current RSV infection when the infant had been infected formerly, and *D*(1) was the potential current RSV infection when the infant had not. Thus, compliers were infants who had a current RSV infection if they had not been infected formerly, *D*(1) = 1, and did not have a current RSV infection if they had been infected formerly, *D*(0) = 0. Always-takers were infants who had a current RSV infection regardless of former RSV infection. Never-takers were infants who did not have a current RSV infection regardless of former RSV infection. Defiers were infants who had a current RSV infection if they had been infected formerly, *D*(0) = 1, and did not have a current RSV infection if they had not been infected formerly, *D*(1) = 0. We assumed that monotonicity held, and thus defiers were excluded from the analysis. Table 1 is a frequency table for former RSV infection (i.e., the IV) and current RSV infection. Among those who had been infected formerly, 20% were not infected, while among those who had not been infected formerly, 34% were infected. Thus, former RSV infection increased the likelihood of current RSV infection. The overall IV strength was approximately 15%. The partial F-test statistic for the proposed IV was 10.62, which is on the boderline of the typical rule of thumb of 10 [19].

**Table 1:** Former and current RSV infection.

	Current RSV infection	
Former RSV infection	Not infected (0)	Infected (1)
Infected (0)	126	31
Not infected (1)	1921	1000

The IPS was defined as the probability of having no former RSV infection. We estimated the IPSs, pr(*Z* = 1 ∣ *X* = *x*), using a logistic regression model with main effects of the covariates. The estimated IPSs ranged from 0.78 to 0.99. We estimated the compliance scores as described before. The estimated compliance scores ranged from −0.45 to 0.61.

Table 2 lists the inference results for the LATE and TLATE at different values of the cutpoint *t*. The cutpoint value ranged from the 5th to the 25th percentiles of the estimated compliance scores, and the actual values of the 5th and 25th percentiles were −0.177 and 0.029, respectively. We estimated the standard error and 95% confidence interval of the point estimate using bootstrapping: for each casual estimate, we obtained 1000 bootstrap replications, calculated the sample standard error of them, and took their 2.5th and 97.5th percentiles. The LATE estimate was 0.004, and the TLATE estimates were from 0.051 to 0.079. For the purpose of comparison, we estimated the ATE, which was 0.077 with a standard error of 0.017. The TLATE at the 25th percentile was 0.079, which is similar to the ATE, but it was not significantly different from 0. We defined the relative efficiency as the variance of the LATE estimator divided by that of the estimator under consideration [27]. Following this definition, the relative efficiency of the TLATE at the 25th percentile was 1.84.

**Table 2:** LATE truncated at different percentiles of the estimated compliance scores: Point estimates, standard errors, and IV strengths. The LATE truncated at the 0th percentile represents the standard LATE.

Percentile	Estimate	IV strength	SE	LB	UB
0.00	0.004	0.146	0.317	−0.620	0.606
0.05	0.072	0.176	0.267	−0.451	0.609
0.10	0.074	0.173	0.268	−0.441	0.665
0.15	0.056	0.194	0.293	−0.390	0.552
0.20	0.051	0.199	0.248	−0.400	0.588
0.25	0.079	0.205	0.233	−0.355	0.582

“SE” is the standard error of the LATE or TLATE estimate. “LB” and “UB” are the lower and upper bounds of the 95% confidence interval.

## Conclusion

Approaches dealing with the positivity issue with the PSs of treatment have been well developed, including the truncation and overlap weights methods [14, 15]. We proposed an IV estimation of truncated LATEs to deal with the problem of weak instruments and the related positivity violation. The proposed method resembles the truncation approach for PS weighting, but it removes the subjects who are very unlikely to be compliers. Our simulation study showed that the relative efficiency of the proposed TLATE increases as the variation of the compliance score increases. Therefore, our method will be useful for data for which the instruments are weak but the compliance score varies considerably across subjects. In our analysis of PRI.DE data, the estimated compliance score varied considerably across infants, and approximately 20% of the subjects had negative compliance scores. The analysis showed that the TLATE estimates were more precise than the standard LATE estimate.

A main limitation of our approach is to lose a part of the study sample by excluding some subjects based on compliance scores, and thus the LATE can be estimated from a smaller population of compliers. The reduced study sample, however, can represent a population that is more desirable for IV analysis because the subjects who are very unlikely to be compliers are removed. In general, the relative population size of compliers estimated from the reduced sample grows as the cutpoint for compliance scores increases. One interesting measure for IV analysis is the effective sample size of compliers, which can be calculated as the product of the sample size and IV strength. Our analysis of PRI.DE data shows that the relative population size as well as the effective sample size can be increased after truncation. The effective sample size based on the untruncated PRI.DE data is 3078 × 0.146 ≈ 450, while that based on the truncated data at the 25th percentile is 3078 × 0.75 × 0.205 ≈ 474.

We used maximum likelihood to estimate a logistic regression model for the IPSs, but other estimation methods can be used. To reduce the impact of model misspecification in parametric modeling, one can use the covariate balancing PS [28]. One could also use nonparametric methods, such as generalized boosting models in which the tuning parameters are selected to optimize covariate balance [29]. Various comparison studies have shown that these methods, when used for PS weighting or doubly robust estimation, work more favorably than maximum likelihood in various simulation scenarios, but their comparative performances depend on the data generating models for the outcome and treatment [30–33].

## Appendix: Proof of Theorem 1

By Bayes’ theorem, *f*^*c*^(*x*) = pr(*U* = 2 ∣ *X* = *x*)*f*(*x*)/pr(*U* = 2). Because pr(*U* = 2 ∣ *X* = *x*) = *δ*(*x*), we have *f*(*x*) = *δ*(*x*)^−1^ pr(*U* = 2)*f*^*c*^(*x*) Then, $\theta_{T}^{c}$ in (4) becomes

$$\begin{array}{ccc} \theta_{T}^{c} & = & \frac{\int \tau (x)1\{\delta (x)>t\}f(x)dx}{\int \delta (x)1\{\delta (x)>t\}f(x)dx},\\ & = & \frac{\int \tau \left(x\right)1\left\{\delta \left(x\right)>t\right\}\delta {\left(x\right)}^{-1}\text{pr}\left(U=2\right)f^{c}\left(x\right)dx}{\int \delta \left(x\right)1\left\{\delta \left(x\right)>t\right\}\delta {\left(x\right)}^{-1}\text{pr}\left(U=2\right)f^{c}\left(x\right)dx},\\ & = & \frac{\int \theta^{c}\left(x\right)1\left\{\delta \left(x\right)>t\right\}f^{c}\left(x\right)dx}{\int 1\left\{\delta \left(x\right)>t\right\}f^{c}\left(x\right)dx}. \end{array}$$ (7)

Eq (7) holds because *θ*^*c*^(*x*) = *τ*(*x*)/*δ*(*x*).
